# Treatment of olecranon fractures in childhood: A systematic review

**DOI:** 10.3389/fped.2022.1046243

**Published:** 2022-11-18

**Authors:** Fernando De Maio, Giulio Gorgolini, Alessandro Caterini, Claudia Luciano, Dario Covino, Pasquale Farsetti

**Affiliations:** Section of Orthopaedics and Traumatology, Department of Clinical Science and Traslational Medicine, University of Rome “Tor Vergata”, Rome, Italy

**Keywords:** olecranon, fracture, children, surgery, upper limb, surgical procedures, surgical treament, pediatric

## Abstract

**Background:**

Literature over the last 20 years provides evidence for a surgical treatment of displaced olecranon fractures in children, this is usually obtained with commonly proposed methods, although there is no general agreement about the best recommended technique.

**Aim:**

Identifying the best surgical technique in displaced olecranon fractures in children and the role of associated fractures in the prognosis of these lesions, by analyzing the most relevant studies on this topic.

**Methods:**

A literature search was performed in MEDLINE database and Scopus database. Articles reporting clinical outcomes of pediatric patients affected by olecranon fractures treated surgically were identified.

**Results:**

The initial search produced 111 studies, with 8 fulfilling the eligibility criteria of our study. Selected articles (2002–2022) included 122 patients overall.

**Conclusion:**

Displaced olecranon fractures, occurring during skeletal growth and surgically treated, generally have good results, although we are unable to recommend the best surgical treatment based on our review. In most cases, they are intra-articular fractures; thus, the overall goal is to get an anatomic reduction that in some cases cannot be obtained by percutaneous techniques. Tension band suture is the preferred device, although it is not recommended in adolescence for the high risk of fixation failure. Associated lesions may affect results.

## Introduction

Fractures of the olecranon are rare and account for 5% of all elbow fractures during skeletal growth (1). These fractures generally occur between 5 and 10 years of age and the most common mechanism of injury is trauma onto either an outstretched hand or a flexed elbow; they are commonly associated with additional fractures of the radial head or the distal part of the humerus. Fractures with displacement greater than 2 mm generally require surgical treatment (2). In 2002, we reported a long-term follow-up study with an average follow-up of 23.8 years, on 39 cases, the majority of which were treated conservatively. We conclude that the long-term prognosis of olecranon fractures in children is related to the anatomic site of the fracture line, to the interfragmentary displacement and to the presence of an associated lesion that represent a negative prognostic factor (3). Classification of these rare lesions is still debated and to the best of our knowledge, there is no universally accepted classification in the literature. Generally, in all the classification systems reported, the possible presence of the intra-articular displacement more than 2 mm and the presence of associated injuries are considered (4, 5). In our study we proposed a classification in 5 types, on the basis of the anatomic site of the fracture line, the inter-fragmentary displacement and the presence of an associated lesion (3). There is general agreement that undisplaced or minimally displaced (less than 2 mm) fractures may be treated conservatively with good results, while displaced fractures need to be treated surgically. The most common methods of treatment proposed in the last 20 years are open reduction and internal fixation (ORIF) with tension band wiring or suture, open or percutaneous screw fixation and ORIF with plate and screws. The aim of our systematic review was to identify the best method of surgical treatment in displaced olecranon fractures in children and the role of associated fractures in the prognosis of these lesions.

## Materials and methods

Inclusion and exclusion criteria were formulated according to the population, intervention, comparator, outcome (PICO) method and are summarized in Table 1 (6). Search strategy and sources of information: Authors of this review (PF, FM, GG, DC, AC, and CL) performed a literature search about the topic by querying Medline database, Scopus and Chocrane Library. The search strategy covers PICO and was performed independently by each author in July 2021. Keywords and Medical Subject Headings (MeSH) terms were identified by a preliminary search and selected by discussion. The search was conducted using the following keywords and their synonyms or MeSH Terms assembled in various combinations to obtain most pertinent articles: olecranon, fractures, children. The following is the list of all of the terms used and the Boolean operators used to combine them: ((“olecran*”[Title]) OR ((“olecranon process/injuries”[MeSH Terms] OR “olecranon process/surgery”[MeSH Terms] OR “olecranon process/therapy”[MeSH Terms]))) AND (“fractur”[All Fields] OR “fractural”[All Fields] OR “fracture's”[All Fields] OR “fractures, bone”[MeSH Terms] OR (“fractures”[All Fields] AND “bone”[All Fields]) OR “bone fractures”[All Fields] OR “fracture”[All Fields] OR “fractured”[All Fields] OR “fractures”[All Fields] OR “fracturing”[All Fields]) AND (“Child”[Mesh] OR “Adolescent”[Mesh] OR “Pediatrics”[Mesh] OR “Child*”[Title] OR “Pediatr*”[Title]).

**Table 1:** Inclusion and exclusion criteria (PICOT).

	Inclusion criteria	Exclusion criteria
Population	- Children Patients (<18aa) affected by olecranon fractures - Patients affected by associated fractures	- Patients who didn’t underwent surgery. - Patient affected by fracture-dislocation of the proximal ulna. - Patients affected by Osteogenesis Imperfecta
Intervention	- Open or percutaneous fixation of fracture site.	- Non surgical techniques with closed reduction without fixation - Non-surgical treatment
Comparison group	- Studies reporting patients treated with different surgical techniques will be compared.	- Not applicable
Outcome	- Studies reporting clinical and radiographic scores	- Not reporting clinical results
Time	- Studies published from 2002 to 2022	- Studies published prior to 2002
Study type	- Original Articles - Clinical Trials - Cohort Studies - Observational Studies - Randomised Control Trials	- Letters - Case reports - Experimental Studies
Language	- English	- Other languages

A publication date filter was applied to select only articles and review articles from the last 20 years (ranging from 2002 to 2022). Language restriction filter was applied to identify only English articles.

The reviewers (PF, FM, GG, DC, AC, and CL) retrieved the data and independently analyzed each selected study; instances of disagreement were resolved by the senior investigator (PF).

The articles were screened for the presence of the following inclusion criteria: pediatric patients affected by olecranon fractures; patients treated with any surgical technique; studies providing an adequate level of evidence, including retrospective studies; availability of full text. The studies were excluded if they provided information regarding: patients affected by Osteogenesis Imperfecta or affected by fracture-dislocation of the proximal ulna; patients treated with non-surgical techniques or with closed reduction without fixation. Letters, Case reports or Experimental Studies and studies not reporting clinical results were also excluded.

## Results

The initial search produced 111 studies. After a first screening, by reading title and abstract and evaluation based on inclusion and exclusion criteria, articles were screened and only 10 studies fulfilled the eligibility criteria of our study. The other studies were excluded for the following reasons: 28 were Case Reports; 28 reported fractures not involving olecranon; 26 were about adult patients; 4 didn't report surgical treatment; 4 reported cases affected by osteogenesis imperfecta; 2 reported less than 5 cases; 2 reported fracture dislocations; 2 reported cases affected by congenital pseudoarthrosis; 2 were experimental studies; 2 reported stress fracture; 1 reported nonunion; 1 didn't report follow-up.

**Figure 1:**
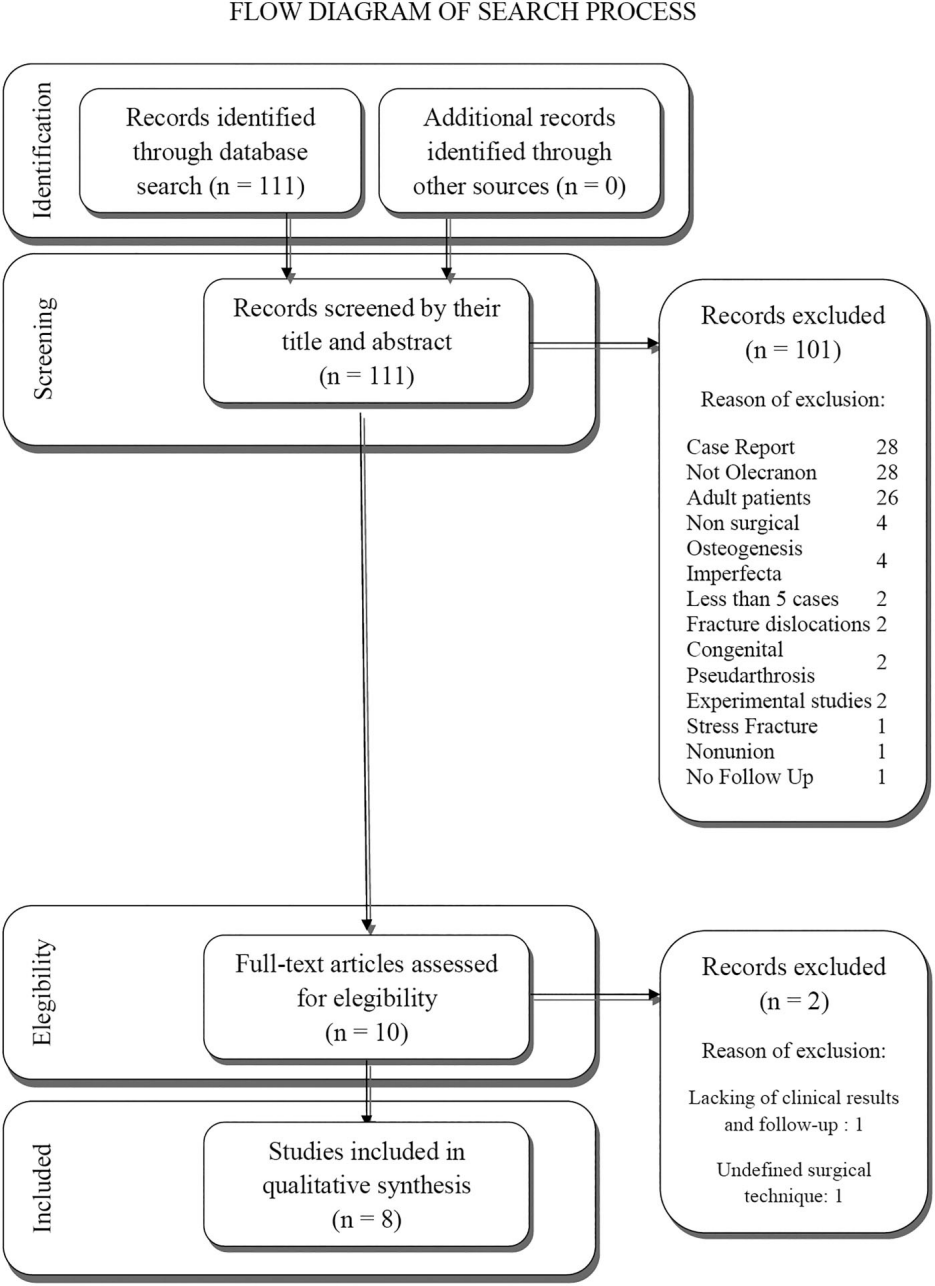
Flow diagram of search process.

After screening the full text of the remaining 10 articles, we excluded 2 more articles which lacked follow-up measure, clinical outcomes and reported unspecified surgical technique. In conclusion, a total of 8 articles were enrolled in the present review (Figure 1 shows the flowchart for study selection). All the selected articles were published from 2002 to 2022 and included 122 patients overall. Table 2 presents a list of the studies, summarizing the number of patients, classification of fracture, associated lesions, age at surgery, surgical technique performed, length of follow-up, results and conclusions.

**Table 2:** This table presents a list of the included studies, summarizing the number of patients, classification of fracture, associated lesions, age at surgery, surgical technique performed, length of follow-up, results and conclusions.

Authors	Year of pubblication	Title	Study type	Number of cases (surgically treated)	Classification of fracture	Associated fractures	Average age at surgery	Surgical treatment	Length of follow-up	Final results
Caterini et al.	2002	Fractures of the olecranon in children. Long-term follow-up of 39 cases.	Retrospective	5	Caterini: 5 types	NO	7.4 years	ORIF cerclage wiring (4 cases) one screw (1 case)	32 years	Good: 4 cases poor: 1 case (inadequate reduction)
Karlsson et al.	2002	Fractures of the olecranon during growth: a 15–25-year follow-up.	Retrospective	11	Horne and Tanzer: 3 types	NO	11 years	ORIF figure-of-eight wiring (6 cases) tension band wiring (2 cases) Rush pin (1 case) CRIF percutaneous pinning (2 cases)	19 years	Excellent: 9 cases good: 2 cases (occasional symptoms)
Gicquel et al.	2003	Surgical technique and preliminary results of a new fixation concept for olecranon fractures in children.	Retrospective	6	Bracq: 3 types	2 (radial head)	10.2 years	CRIF two threaded pins (percutaneous)	14 months	Excellent: 5 cases good: 1 case (limited ROM—10° flexion + associated radial head fracture)
Gortzak et al.	2006	Pediatric olecranon fractures: open reduction and internal fixation with removable Kirschner wires and absorbable sutures.	Retrospective	6	Intra-articular displaced fractures (no specific classification)	3 (humeral condyle—1, radial head—2)	9.7 years	ORIF removable K wires and figure-of-eight suture	13 months	Excellent: 5 cases good: 1 case (limited ROM—10° extension)
Corradin et al.	2016	Outcome of isolated olecranon fractures in skeletally immature patients: comparison of open reduction and tension band wiring fixation versus closed reduction and percutaneous pinning.	Retrospective	22	AO PCCF classification: 3 types	NO	10.5 years	ORIF tension band wiring (10 cases) CRIF percutaneous 4.5mm cannulated screw (12 cases)	17.9 momths	Quick DASH score: 1.82/3.42 no statistical significant difference between the 2 groups (limited ROM—20° extension—4 patients)
Kim et al.	2017	Early range of motion exercise in pediatric patients with olecranon fractures treated with tension band suture with double loops and double knots.	Retrospective	12	AO PCCF classification: 3 types	2 (radial head)	10.6 years	ORIF K wires and figure-of-eight suture early ROM	12 months	Perfect MEPS: 12 cases
Perkins	2018	Olecranon fractures in children and adolescents: outcomes based on fracture fixation.	Retrospective	46	Intra-articular displaced fractures (no specific classification)	5 (radial head—4 distal radius—1)	12.3 years	ORIF tension band wiring (17 cases) tension band suture (29 cases)	9.1 months	Revision fixation (TBW: 1 case TBS: 4 cases) children eavier than 50 kg having higher rates of fixation failure with TBS
Li et al.	2022	Short-Term Outcomes of Herbert Screw Fixation for Isolated Olecranon Fractures in Children: A Single-Institution Retrospective Study.	Retrospective	14	Mayo: 3 types	NO	11.3 years	CRIF two 3 mm Herbert screw (percutaneous)	11.9 months	Quick DASH score: 1.58

## Discussion

Surgical treatment of displaced olecranon fractures in children is still debated. On the contrary, conservative treatment is usually adopted in non-displaced or minimally displaced fractures with good results. Generally, the majority of authors considered minimally displaced olecranon fractures when the interfragmentary gap is more that 2–3 mm.

In this systematic review, we analyzed the clinical and radiological results obtained in 122 children treated surgically, from eight clinical and radiological studies published in the last 20 years. All these studies were retrospective and the majority of them had a short-term follow-up. The surgical techniques commonly reported are open or closed reduction followed by internal fixation using various devices. Tension band wiring (TBW), tension band suture (TBS) and cerclage techniques are the most common devices used after open reduction while screws or pins are generally applied percutaneously after closed reduction.

Usually, the classification systems are proposed to help guide treatment; however, in regards to olecranon fractures in pediatric patients, several classifications have been reported, without any demonstrating superiority over the others. The adopted classifications in our review are the following: Caterini et al. who proposed five different fracture types (3), on the basis of the anatomic site of the fracture line (7), interfragmentary displacement and presence of an associated lesion (5); Horne and Tanzer, who proposed three types, depending on location of the fracture on the olecranon (8); AO PCCF, based on the morphology of the fracture (9) and Mayo classification (10) in three types, described for adults fractures, based on fracture displacement and elbow stability (11). In two papers no specific classification is reported; the authors had surgically treated all intra-articular displaced olecranon fractures. These data confirm that, there is still no classification commonly adopted for olecranon fractures in children that suggests the best treatment to adopt.

The majority of papers included in the review are short-term follow-up studies. Gicquel et al. reported the preliminary results of a new percutaneous fixation technique to stabilize six olecranon fractures using two threaded pins introduced by a minimal skin incision with a divergent orientation. Only in two cases, the interfragmentary displacement was more than 2 mm. The authors observed excellent result in all patients but one, which was associated with a radial head fracture and mild limited range of motion of the elbow was present. They concluded that their technique can be used routinely because of its effectiveness and simplicity (12). Recently Li et al. reported another short-term follow-up study on 14 cases treated percutaneously using two cannulated Herbert screws with a different direction (13). All the fractures included in the study had a displacement more than 4 mm that were closed reduced, before percutaneous fixation. The authors observed good functional and radiological results in all patients, evaluated with a quickDASH scoring system. The authors strongly recommend the percutaneous technique, to avoid skin complications and hardware irritation causing persistent joint pain, requiring hardware removal. Moreover they underlined the ease of screws removal performed by small incisions. On the contrary, other authors prefer to perform an open reduction; Gortzak et al. in 2006 (14) and more recently Kim et al. in 2017 (15), reported 6 and 12 olecranon fractures respectively, treated by open reduction and internal fixation with two K-wires and figure-of-eight suture. Both papers reported excellent results at an average of 1 year after treatment, except in one case in which the authors observed a limited elbow extension of 10°. Gortzak et al., suggest leaving the two Kirschner wires out of the skin to perform a quick removal of the devices after fracture healing. The authors emphasize their technique that avoid a reoperation for hardware removal. Kim et al., instead emphasize the early range of motion exercise after stabilization fracture performed by tension band suture with double loops and knots (15). Perkins et al. reported 46 olecranon fractures in children and adolescents comparing 17 patients treated by open reduction and tension band wiring and 29 patients treated by open reduction and tension band suture. The authors, who report the largest series of olecranon fractures included in our review, concluded that tension band suture is contraindicated in patients weighting more than 50 kg; in fact, they observed their failures in older and heavier patients (4 cases) (16). Corradin et al. reported a comparison of open reduction and tension band wiring fixation performed in 10 cases versus closed reduction and percutaneous screw fixation performed in 12. The authors, while reporting a difference regarding the quickDASH score at follow-up between the two groups (1.82 in the open series versus 3.42 in the closed series), concluded that no statistically significant differences were present between the two groups, with equally acceptable clinical and radiological final results and similar rate of complications (17).

Only two papers with a long-term follow-up have been published in the last 20 years. Caterini et al. reported only 5 cases of surgically treated patients and concluded that the long-term prognosis of olecranon fractures in children is related to the anatomic site of the fracture line, to the interfragmentary displacement and to the presence of an associated lesion. They observed only one case with poor result related to an inadequate reduction and fixation (3). Karlsson et al. reported 11 olecranon fractures surgically treated and observed that none of their patient developed nonunion or elbow osteoarthritis, therefore they conclude that olecranon fractures during growth have an excellent long-term results (18).

In four studies of our review associated lesions are reported, most of them are radial head fractures that can affect the final result (3, 14).

In conclusion, based on our review, displaced olecranon fractures occurring during skeletal growth surgically treated with various techniques generally have good results, although we are unable to recommend the best surgical treatment to perform. However, we believe that since they are intra-articular fractures in the majority of cases, the overall goal is to get an anatomic reduction that in some cases cannot be obtained using a percutaneous technique. Regarding the devices, tension band suture is preferred but remains contraindicated in adolescence for the high risk of fixation failure. Associated lesions may affect the final result.

## Data Availability

The original contributions presented in the study are included in the article/Supplementary Material, further inquiries can be directed to the corresponding author/s.
